# Does allergen immunotherapy impact the susceptibility and severity of COVID‐19?

**DOI:** 10.1002/clt2.12247

**Published:** 2023-04-23

**Authors:** Yin Wang, Huan Chen, Xiang Dong, Hao Chen, Hui‐ling Liang, Ya‐qi Yang, Yan‐dan Chen, Rong‐fei Zhu, Ya‐dong Gao

**Affiliations:** ^1^ Department of Allergy Tongji Hospital Tongji Medical College Huazhong University of Science and Technology Wuhan China; ^2^ Department of Otolaryngology‐Head and Neck Surgery and Allergy Central Hospital of Huangshi City Huangshi China; ^3^ Department of Allergology Zhongnan Hospital of Wuhan University Wuhan China

To the Editor,

1

Allergic asthma (AA) and allergic rhinitis (AR) might be protective against SRAS‐CoV‐2 infection and progress to severe disease of coronavirus disease 2019 (COVID‐19).^1^ COVID‐19 vaccination was safe and well tolerated in patients receiving allergen immunotherapy (AIT),^2^, ^3^ and the adherence to subcutaneous immunotherapy (SCIT) was not affected during COVID‐19 pandemic.^4^ AIT induces allergen‐specific tolerance by attenuating allergen‐specific T helper 2 (Th2) cells and inducing regulatory T and B cells and interleukin (IL)‐10.^5^ We hypothesized that AIT may have a protective impact on the susceptibility and severity of COVID‐19.

In December 2022, China ended its ‘Zero‐COVID’ policy and more than 70% of the population got infected with SARS‐CoV‐2 within one month. We conducted an online WeChat questionnaire between 3 January and 10 January 2023 in order to investigate the infection and hospitalization rates and symptom duration of COVID‐19 in patients with AR and/or AA receiving SCIT with house‐dust mite (HDM) extract. The patients with AR and AA receiving HDM‐SCIT were from three allergy centers (Tongji Hospital, Zhongnan Hospital, and Central Hospital of Huangshi City) in Hubei province, China. In this study, HDM‐induced AR was diagnosed according to ARIA (allergic rhinitis and its impact on asthma): typical symptoms of nasal pruritus, sneezing, rhinorrhea, and nasal congestion in combination with evidence of HDM sensitization (positive skin prick test and/or specific IgE).^6^ HDM‐associated AA was diagnosed according to GINA (global initiative of asthma)^7^ and the evidence of HDM sensitization. Since the aim of this study was to investigate the effect of AIT on COVID‐19, only those patients with AR and AA receiving HDM‐SCIT were invited to participate in this study. In the three allergy centers, both *Dermatophagoides pteronyssinus* extract (Alutard SQ Der p, ALK‐Abelló, Hørsholm, Denmark) and Der p/*Dermatophagoides farinae* (Der f) extract (NovoHelisen Depot [NHD]; Allergopharma, Reinbek, Germany) were used for HDM‐SICT in AR and AA patients. The questionnaire was designed by the authors with the App Wenjuanxin, and a two‐dimensional barcode was generated and dispensed in the WeChat group of AR and AA patients receiving HDM‐SCIT. The data of questionnaire were also collected with the App Wenjuanxin.

The relatives of these SCIT patients, who did not receive SCIT, were also surveyed with the same questionnaire and divided into two groups: allergy group, when they did not report any of physician‐diagnosed allergic diseases, including AR, AA, food allergy, and atopic dermatitis, and non‐allergy group, when they did not report a history of any aforementioned allergic diseases. The study was approved by the Medical Ethic Committee of Tongji Hospital of Huazhong University of Science and Technology (Approval Number: TJ‐IRB20230204). The informed consent was waived since the voluntary nature of responding to the questionnaire.

A total of 1246 patients receiving SCIT and 1078 of their relatives (370 allergic and 708 non‐allergic) responded to the questionnaire. Patients with SCIT were generally younger than allergy and non‐allergy groups. The proportion of male was higher in patients with SCIT than allergy and non‐allergy groups. About 82.4% of the patients with SCIT were diagnosed with AR, only 5.3% were asthmatics, and the rest were AR with asthma (12.3%). The average duration of SCIT was 1.4 ± 1.3 years. Patients with SCIT had a lower proportion of both at least one dose and completed three doses of COVID‐19 vaccines when compared to allergy and non‐allergy groups (*p* = 0.000) (Table S1).

Most respondents had been infected with SARS‐CoV‐2. HDM‐SCIT was associated with a lower infection rate (78.6%) compared to allergy (81.4%) and non‐allergy groups (81.5%) (*p* < 0.0001) (Figure 1 and Table S2). The duration of COVID‐19 symptoms was shorter in the SCIT group (5.7 ± 4.0 days) than the allergy group (7.0 ± 4.5 days, *p* = 0.000) and the non‐allergy group (7.7 ± 4.4 days, *p* = 0.000) (Figure 1 and Table S2). The hospitalization rate was 0.4% in the SCIT group, which was significantly lower than that in the non‐allergy group (1.73%) (*p* = 0.008) (Figure 1 and Table S2).

**FIGURE 1:**
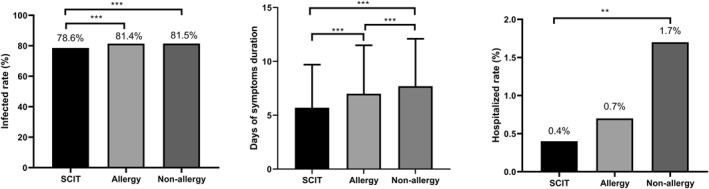
The infection rates, days of symptom duration, and hospitalized rates of COVID‐19 in SCIT, allergy and non‐allergy groups. **p < 0.01; ***p < 0.001.

We then performed a two‐to‐one matching of the SCIT group with allergy and non‐allergy groups to adjust age, sex, and vaccination difference between the three groups. As shown in Table 1, 396 patients with SCIT and 199 patients in allergy and non‐allergy groups were included in the final analysis. The standardized mean differences for age, sex, and COVID‐19 vaccination status were 0.000, 0.000, and 0.005, suggesting that these parameters were well matched among the three groups. However, the difference of infection rate, duration of symptoms, and hospitalization rate among the three groups disappeared after matching (Table 1).

**TABLE 1:** Infection rate of COVID‐19 in three groups after matching for age, sex, and vaccination status by propensity score weighting.

Characteristics	SCIT(*n* = 396)	Allergy(*n* = 199)	Non‐allergy(*n* = 199)	SMD
Age (years)	23.5 ± 16.7	23.5 ± 14.4	23.5 ± 12.2	0.000
Gender (male), *n* (%)	171 (43.2)	86 (43.2)	86 (43.2)	0.000
COVID‐19 vaccination, *n* (%)				0.005
Unvaccinated	33 (8.3)	17 (8.5)	17 (8.5)	
Vaccinated	363 (91.7)	182(91.5)	182 (91.5)	
Infection rate, *n* (%)				0.057
Uninfected	85 (21.5)	36 (18.1)	37 (18.6)	
Infected	311 (78.5)	163 (81.9)	162 (81.4)	
Duration of symptoms (day)	7.0 ± 4.9	6.8 ± 4.5	7.2 ± 4.7	0.049
Hospitalized, *n* (%)	2 (0.7)	1 (0.6)	2 (1.2)	0.039

Moreover, we found that patients receiving 6–12 months SCIT had a shorter duration of symptoms caused by SARS‐CoV‐2 infection than those in the SCIT course <6 months and those receiving SCIT >12 months, even though only one fourth of them completed three doses of COVID‐19 vaccines (Table 2). The duration of SCIT has no impacts on both infection and hospitalization rate (Table 2).

**TABLE 2:** SARS‐CoV‐2 Infection in SCIT patients.

Characteristic	<6 months(*n* = 99)	6–12 months(*n* = 697)	>12 months(*n* = 450)	*p* Value
Age (years)	18.0 ± 14.6	15.8 ± 12.3	20.3 ± 14.4	0.000
Gender (male), *n* (%)	53 (53.5)	422 (60.5)	276 (61.3)	0.348
COVID‐19 vaccination, *n* (%)				0.006
Unvaccinated	10 (10.1)	95 (13.6)	34 (7.6)	
Vaccinated dose	89 (89.9)	602 (86.4)	416 (92.4)	
1	5 (5.1)	33 (4.7)	7 (1.6)	
2	52 (52.5)	388 (55.7)	237 (52.7)	
3	32 (32.3)	173 (24.8)*	164 (36.4)#	
4	0 (0)	8 (1.2)	8 (1.7)	
Infected, *n* (%)	72 (72.7)	541 (77.6)	366 (81.3)	0.110
Duration of symptoms (day)	6.7 ± 4.5	5.3 ± 3.6*	6.2 ± 4.3#	0.000
Hospitalized, *n* (%)	1 (1.0)	2 (0.28)	1 (0.22)	0.573

*Note*: *p = 0.003, <6 months versus 6–12 months; #*p* = 0.001, 6–12 months versus >12 months.

A lower expression of angiotensin converting enzyme 2 (ACE2) in airway epithelia^8^ may contribute to the protecting effect of type 2 inflammation against SARS‐CoV‐2 infection and severe COVID‐19.^9^ This study revealed an almost same infection rates among allergic and non‐allergic individuals after matching for age, sex, and COVID‐19 vaccination status, suggesting that the ACE2 expression level may have a minimal effect on Omicron infection. Actually, patients with SCIT were younger than allergy and non‐allergy groups, which may contribute to the slightly lower infection rate in this group than allergy and non‐allergy groups before matching, since it has been previously reported that younger age was associated with a lower rate of SARS‐CoV‐2 infection.^1^ In addition, repeated allergen stimulation during SCIT in HDM‐sensitized individuals may elicit a strong T cell response with ability to cross‐react with SARS‐CoV‐2, as demonstrated in silico analysis,^10^ which may also protect patients with SCIT from infection. The proportion with three doses COVID‐19 vaccines was significantly lower in patients with SCIT albeit SCIT was reported to dampen immune responses to SASR‐CoV‐2 vaccines.^11^ Nevertheless, the infection rate of SARS‐CoV‐2 was almost same in three groups after matching for age, sex, and vaccination status, suggesting that these parameters may be the dominant factors for the susceptibility to Omicron strain when compared to SCIT or allergy. We also observed a shorter duration of symptoms due to SARS‐CoV‐2 infection in those receiving 6–12 months HDM‐SCIT compared to those receiving <6 months and >12 months HDM‐SCIT, consistent with previous studies showing the immune responses to SCIT reach a peak during 6–12 months.^5^ EAACI stated recently in a position paper that AIT and COVID‐19 immune responses do not seem to interfere negatively, and AIT patients might even benefit from COVID‐19 vaccination.^12^

Our study has limitations. First, the high COVID‐19 vaccination rate in the whole population may cover up the protecting effect of SCIT; second, not all patients receiving SCIT in the three centers were included in the study, only those using the WeChat App and within the special WeChat group of SCIT patients may respond to the questionnaire, and the response to the questionnaire may also be influenced by the frequency and skills of using this app; finally, the allergy and non‐allergy status were self‐reported by the participants without verification by physicians.

In conclusion, our results for the first time demonstrated that SCIT may have a slight protective effect against SARS‐CoV‐2 infection, especially immediately after completing the dose‐escalation phase; however, this effect may be covered up by COVID‐19 vaccination.

## AUTHOR CONTRIBUTIONS

Ya‐dong Gao, Rong‐fei Zhu, and Yan‐dan Chen conceived the study, Yin Wang and Huan Chen designed the questionnaire and collected data. Xiang Dong, Hao Chen, Ya‐qi Yang, and Hui‐ling Liang dispensed the questionnaire and monitored the survey. Rong‐fei Zhu analyzed the data and Ya‐dong Gao wrote the manuscript. All authors contributed to the final review.

## CONFLICT OF INTEREST STATEMENT

The authors declare that they have no conflicts of interest.

## FUNDING INFORMATION

None.

## Supporting information

Supporting Information S1
