# Supplementary material for: Does allergen immunotherapy impact the susceptibility and severity of COVID‐19?
Source: Clin Transl Allergy. 2023 Apr 23;13(4):e12247. doi: 10.1002/clt2.12247 (PMC10123383; doi:10.1002/clt2.12247)
Supplement: Supplementary file 1 — Supporting Information S1 [file CLT2-13-e12247-s001.doc]

**Table S1. Demographic characteristics of participants.**

| **Characteristic** | **SCIT**  **(n=1246)** | **Allergy**  **(n=370)** | **Non-allergy**  **(n=708)** | ***P* value** |
| --- | --- | --- | --- | --- |
| Age (years) | 17.6 ± 13.4 | 32.2 ± 15.1* | 41.6 ± 18.2^#&^ | 0.000* |
| Gender (male), n (%) | 751 (60.3) | 179 (48.4)* | 300 (42.4)^#^ | 0.000* |
| Disease, n (%) |  |  |  |  |
| AR | 1027 (82.4) | 267 (72.2) | - |  |
| AS | 66 (5.3) | 43 (11.6) | - |  |
| AR&AS | 153 (12.3) | 60 (16.2) | - |  |
| AIT duration (years) | 1.4 ± 1.3 | - |  |  |
| **COVID-19 vaccination, n (%)** |  |  |  | 0.000 |
| Unvaccinated | 130 (10.4) | 18 (4.9)* | 47 (6.6)# |  |
| Vaccinated dose | 1116 (89.6) | 352 (95.1) | 661 (93.4) |  |
| 1 | 41 (3.3) | 5 (1.35) | 1 3 (1.8) |  |
| 2 | 672 (53.9) | 82 (22.2) | 115 (16.2) |  |
| 3 | 387 (31.1) | 255 (68.9)* | 527 (74.4)# |  |
| 4 | 16 (1.3) | 10 (2.7) | 6 (0.8) |  |

**P*=0.000, SCIT vs. Allergy; #*P*= 0.000, SCIT vs. Non-allergy; &*P*=0.000, Allergy vs. Non-allergy.

**Table S2. Infection rate and symptoms of COVID-19 in participants.**

| **Characteristics** | **SCIT (n=1246)** | **Allergy**  **(n=370)** | **Non-allergy**  **(n=708)** | ***P* value** |
| --- | --- | --- | --- | --- |
| **Infection, n (%)** |  |  |  | 0.000 |
| Uninfected | 267 (21.4) | 69 (18.7) | 131 (18.5) |  |
| Infected | 979 (78.6) | 301 (81.4)* | 577 (81.5)# |  |
| RT-PCR + | 166 (13.3) | 51 (13.8) | 67 (9.5) |  |
| Antigen+ | 351 (28.2) | 138 (37.3) | 234 (33.1) |  |
| RT-PCR+ and antigen+ | 56 (4.5) | 13 (3.5) | 36 (5.1) |  |
| Symptomatic without RT-PCR of antigen test | 406 (32.6) | 99 (26.8) | 240 (33.9) |  |
| **Symptoms in infectants,**  **n (%)** |  |  |  |  |
| Asymptomatic | 15 (1.5) | 6 (2.0) | 3 (0.5) | 0.086 |
| Fever | 802 (81.9) | 246 (81.7) | 495 (85.8) |  |
| Fatigue | 324 (33.1) | 145 (48.2) | 385 (66.7) |  |
| Cough | 473 (48.3) | 219 (72.8) | 366 (63.4) |  |
| Sore throat | 333 (34.0) | 136 (45.2) | 304 (52.7) |  |
| Anosmia, ageusia | 187 (19.1) | 91 (30.2) | 157 (27.2) |  |
| Blocked nose, running nose | 456 (46.6) | 147 (48.8) | 255 (44.2) |  |
| Conjunctivitis | 38 (3.9) | 13 (4.3) | 6 (1.0) |  |
| Myalgia | 296 (30.2) | 138 (45.9) | 206 (35.7) |  |
| Diarrhea | 120 (12.3) | 38 (12.6) | 46 (8.0) |  |
| Erythra | 3 6 (3.7) | 22 (7.3) | 17 (2.95) |  |
| **Duration of symptoms** | 5.7 ± 4.0 | 7.0 ± 4.5* | 7.7 ± 4.4#& | 0.000 |
| **Hospitalized, n (%)** | 4 (0.4) | 2 (0.7) | 10 (1.7)^Δ^ | 0.030 |

**P*=0.000, SCIT vs. Allergy; #*P* = 0.000, SCIT vs. Non-allergy; &*P*=0.000, Allergy vs. Non-allergy; ^Δ^P= 0.008, SCIT vs. Non-allergy.
